# Origin of the blueshift of photoluminescence in a type-II heterostructure

**DOI:** 10.1186/1556-276X-7-654

**Published:** 2012-11-27

**Authors:** Masafumi Jo, Mitsuru Sato, Souta Miyamura, Hirotaka Sasakura, Hidekazu Kumano, Ikuo Suemune

**Affiliations:** 1 RIES, Hokkaido University, Kita-21, Nishi-10, Sapporo, 001-0021, Japan; 2 Japan Society for the Promotion of Science (JSPS), 1-8, Chiyoda-ku, Tokyo, 102-8472, Japan; 3 Japan Science and Technology Corporation (CREST), Saitama, 332-0012, Japan; 4 Photonic Materials Unit, National Institute for Materials Science, 1-2-1 Sengen, Tsukuba, Ibaraki, 305-0047, Japan

**Keywords:** Quantum well, type-II, Blueshift, Excitons, GaSb, GaAs, Photoluminescence, 71.35.-y: Excitons, 78.55.Cr: Photoluminescence of III-V semiconductor, 81.15.Hi: Molecular beam epitaxy

## Abstract

Blueshifts of luminescence observed in type-II heterostructures are quantitatively examined in terms of a self-consistent approach including excitonic effects. This analysis shows that the main contribution to the blueshift originates from the well region rather than the variation of triangular potentials formed in the barrier region. The power law for the blueshift, *ΔE*_*PL*_ ∝ *P*_*laser*_^*m*^, from *m* = 1/2 for lower excitation *P*_laser_ to *m* = 1/4 for higher excitation, is obtained from the calculated results combined with a rate equation analysis, which also covers the previously believed *m* = 1/3 power law within a limited excitation range. The present power law is consistent with the blueshift observed in a GaAsSb/GaAs quantum well.

## Background

Interest has recently been increasing in type-II heterostructures in which electrons and holes are separated in adjacent different materials, thereby forming spatially indirect excitons [1-9]. The wavefunction of the indirect exciton is significantly extended in space compared with that of a direct exciton in a type-I system where both electrons and holes are confined in the same layer, which allows large controllability of the wavefunction distribution. In addition, the long radiative lifetime originating from spatially indirect recombination is attractive for applications such as optical memories [10,11].

The separation of charge carriers in a type-II system also induces electrostatic potential (Hartree potential), which causes band bending and a resultant significant change in the exciton wavefunction distribution. Experimentally, this band-bending effect has been observed in power-dependent photoluminescence (PL) measurements, in the blueshift of PL peaks with increasing excitation power [1,2,5,6,12]. The mechanism of this effect has been discussed in terms of a triangular potential model in which photogenerated electrons and holes form a dipole layer, creating a triangular-like potential at the interface [1]. With increasing excitation power, the potential becomes steeper and the quantization energy increases, giving rise to a blueshift of the recombination energy. Following this model, the blueshift is proportional to the cube root of the excitation power, which has been generally accepted for the characterization and distinction of type-II heterostructures.

However, detailed examinations of the observed power dependency sometimes show deviations from the cube root of power law. This is especially noticeable when the excitation power dependence is examined over a wide range. Here, we reexamine the characteristic blueshift in a type-II system using a GaAsSb/GaAs quantum well (QW). We observe that the blueshift does not obey a single-exponent power law, but instead tends to saturate with increasing excitation power. This is analyzed on the basis of a self-consistent band calculation. The dominant contribution to the blueshift originates from the variation of the QW energy level rather than the variation of the triangular potentials formed in the barrier layers, which modifies the cube-root power law.

## Methods

The sample containing a 6-nm GaAsSb QW was grown on a GaAs(001) substrate by MOMBE. The Sb composition of GaAsSb was set at 8%, which was confirmed by XRD. At this Sb concentration, the band lineup between GaAs and GaAsSb becomes a type-II alignment with holes confined in the GaAsSb well [13,14]. The excitation power dependence of the PL was measured at 23 K using the 633 nm line of a He-Ne laser with an intensity range of 1 to 100 W cm^-2^. The incident beam was chopped using an optical chopper to avoid heating.

## Results and discussion

**Figure 1:**
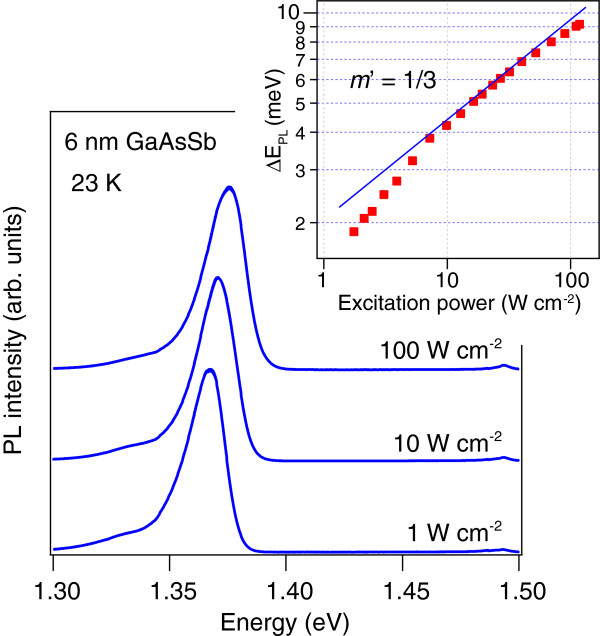
**Low-temperature PL spectra of a 6-nm GaAsSb QW at different excitation densities.** The inset plots the PL peak energy shift as a function of the excitation power density fitted with the conventional cube-root power law.

Figure 1 shows the normalized PL spectra of the sample as a function of excitation power. The spectra show a typical blueshift with increasing excitation power. The shift of the PL peak energy is summarized in the inset, which clearly shows that the cube-root power law only holds within a limited range. The power exponent is greater than 1/3 at low excitation, then decreases and becomes smaller than 1/3 at high excitation.

To elucidate the origin of the characteristic blueshift, let us start with a semi-quantitative analysis of the band bending in a type-II system. We will deal with a single QW structure, and study the band bending effect numerically using a simple one-band model for both the electron and the heavy hole. The excitonic effect is not taken into account at this stage. The one-particle effective mass Schrödinger equations are given by

(1) $$\left[-\frac{\hbar^{2}}{2m_{\mathrm{iz}}}\frac{{\text{d}}^{2}}{\text{d}z^{2}}+V_{i}\left(z\right)+\phi \left(z\right)\right]\psi_{i}\left(z\right)=E_{i}\psi_{i}\left(z\right)\text{,}$$

where *i* = *e* (electron) or *h* (hole), *m*_*iz*_ is the carrier effective mass in the growth direction *z*, *V*_*i*_(*z*) the heterostructure potential and φ(*z*) the self-consistent Hartree potential induced by the spatial separation of the charged carriers. The Hartree potential is obtained from Poisson’s equation,

(2) $$\frac{{\text{d}}^{2}}{\text{d}z^{2}}\phi \left(z\right)=-\frac{e^{2}}{\varepsilon \varepsilon_{0}}\;\left\{n_{h}\left(z\right)-n_{e}\left(z\right)\right\}\text{,}$$

in which ε is the dielectric constant, ε_0_ is the permittivity of vacuum, and *n*_*i*_ is the carrier density determined by the normalized wavefunctions ψ_*i*_(*z*):

(3) $$n_{i}\left(z\right)=n_{s}{\left|\psi_{i}\left(z\right)\right|}^{2}\text{.}$$

The sheet charge density *n*_s_ is a parameter which is an increasing function of the excitation power. Equations 1, 2 and 3 are solved iteratively until they converge.

**Figure 2:**
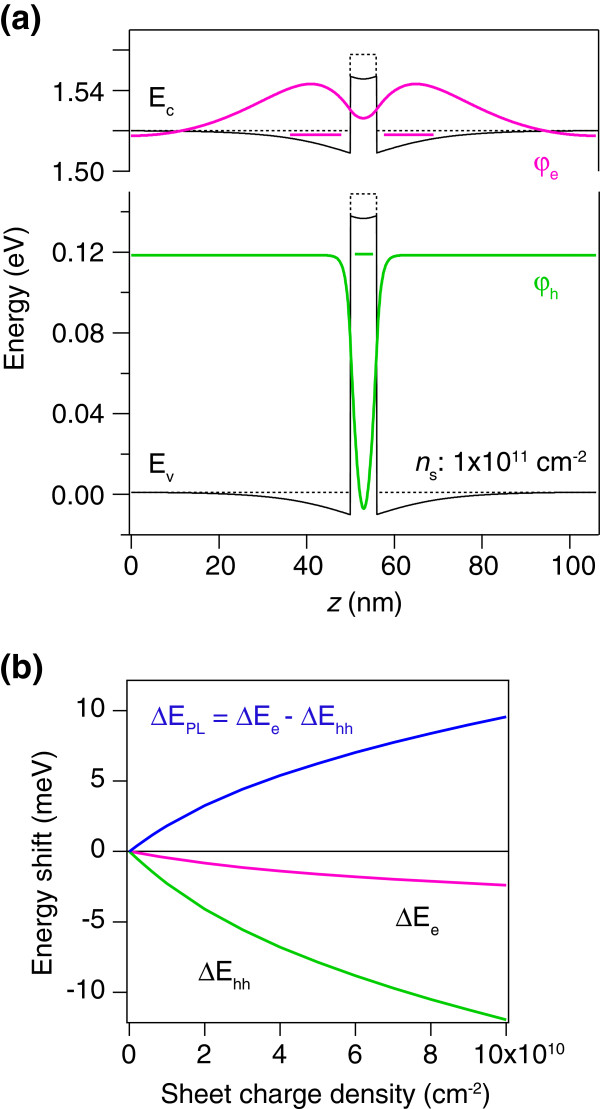
**One-band model calculation for a type-II QW.** (**a**) Calculated band diagram of a 6-nm GaAsSb/GaAs QW for a sheet charge density of 1 × 10^11^ cm^-2^: self-consistent potential (solid lines) and flat-band potential (dashed lines). Electron and heavy-hole wavefunctions with their eigenenergies are also plotted. (**b**) Calculated energy shift of the ground state for the electron (ΔE_e_) and the heavy-hole (ΔE_hh_) with respect to the flat-band condition. The transition energy shift (ΔE_PL_) is given by the difference between the two energy shifts.

Figure 2a shows the calculated band diagram of the GaAsSb/GaAs QW for a sheet charge density *n*_s_ = 1 × 10^11^ cm^-2^. The self-consistent potential is shown by the solid lines, and the flat-band potential by the dashed lines. Electron and heavy-hole wavefunctions with their eigenenergies under the bending band are also plotted in Figure 2a. The parameters used in the calculation are summarized in Table 1. In this heterostructure, holes are confined in the GaAsSb well, whereas electrons are loosely bound to the triangular potential wells formed at the GaAsSb/GaAs interfaces. The ground state energy of the electron under the bending band is lower than that under the flat band because of the attractive Hartree potential, which results in a redshift of the transition energy. However, the hole ground state is also pushed down by the band bending, which leads to a blueshift. The total transition energy is thus dependent on two competing shifts.

**Table 1:** Material parameters used for the calculation of a GaAsSb/GaAs QW

	**GaAs**	**GaAs**_**0.92**_**Sb**_**0.08**_
m_ez_	0.067	0.065
m_eρ_	0.067	0.065
m_hz_	0.35	0.342
m_hρ_	0.11	0.105
ε	13.1	13.3
V_e_ (meV)	38	
V_h_ (meV)	148	

Band offsets are calculated from Ref. [13]. Dielectric constants are from [15]. Other parameters are taken from [16].

To see how the transition energy shifts with the excitation, we calculated the energy shifts of the electron and heavy hole as a function of the sheet charge density Figure 2b. The energy shift of the optical transition, ΔE_PL_, is given by the difference between the two energy shifts: ΔE_PL_ = ΔE_e_ - ΔE_hh_. As the sheet charge density increases, both the electron and heavy-hole energy levels monotonically decrease due to the increasing Hartree potential. Furthermore, the heavy-hole energy shift is *always* larger than the electron energy shift. As a result, the transition energy shift ΔE_PL_ shows a blueshift with increasing excitation. Indeed, this trend is generally true for a type-II structure; the confined carrier (here the hole) is more susceptible to the Hartree potential. This response is partly because the potential well for the electrons is formed at the skirt of the Hartree potential, while holes are affected by the peak height of the Hartree potential. In addition, an increase in the steepness of the triangular well for the electrons raises the quantization energy, compensating for the energy decrease due to the increased well depth.

Having confirmed that the blueshift is mainly caused by the energy shift of the hole in the well, we consider the power dependency of the peak shift. To the zero-order approximation, the hole energy change is proportional to the depth of the Hartree potential, which is, in turn, proportional to the sheet charge density if the holes and electrons are completely separated. The calculated energy shift in Figure 2b shows sublinear dependence on the sheet charge density, indicating that the distribution of electrons and holes under the bending band plays an important role. For more quantitative evaluation, especially at low excitation regimes, it is necessary to include excitonic effects in the calculation. We performed a calculation of the exciton energy under the bending band following [17]. The Schrödinger equation for the exciton is

(4) $$\begin{array}{l} {}[-\frac{\hbar^{2}}{2m_{\rho }}\frac{1}{\rho }\frac{\partial }{\partial \rho }\rho \frac{\partial }{\partial \rho }-\frac{\hbar^{2}}{2m_{\mathrm{ez}}}\frac{\partial^{2}}{\partial {z_{e}}^{2}}-\frac{\hbar^{2}}{2m_{\mathrm{hz}}}\frac{\partial^{2}}{\partial {z_{h}}^{2}}\\ \;+{\tilde{V}}_{e}\left(z_{e}\right)+{\tilde{V}}_{h}\left(z_{h}\right)-\frac{e^{2}}{4\pi \varepsilon \varepsilon_{0}}\frac{1}{\sqrt{\rho^{2}+{\left(z_{e}-z_{h}\right)}^{2}}}]\\ \psi (\rho ,z_{e},z_{h})=E\psi (\rho ,z_{e},z_{h})\text{.} \end{array}$$

Here, 1/*m*_*ρ*_ = 1/*m*_*eρ*_ + 1/*m*_*hρ*_ and is the in-plane reduced mass, and ρ is the in-plane electron–hole distance. The Hartree potential is included in the calculation through the modified heterostructure potential V∼izi=Vi(z)∓ϕzi, where *i* = *e* (electron) corresponds to the upper sign, and *h* (hole) to the lower sign. For simplicity, we ignore the spatially dependent dielectric screening in Equation 4. To solve Poisson’s equation, the carrier density *n*_i_ is obtained by

(5) $$n_{e,h}\left(z_{e,h}\right)=n_{s}\int_{0}^{\infty }\text{d}\rho \int_{-\infty }^{\infty }\text{d}z_{h,e}2\pi \rho {\left|\psi \left(\rho ,z_{e},z_{h}\right)\right|}^{2}\text{.}$$

Again, the sheet charge density *n*_s_ is a parameter.

**Figure 3:**
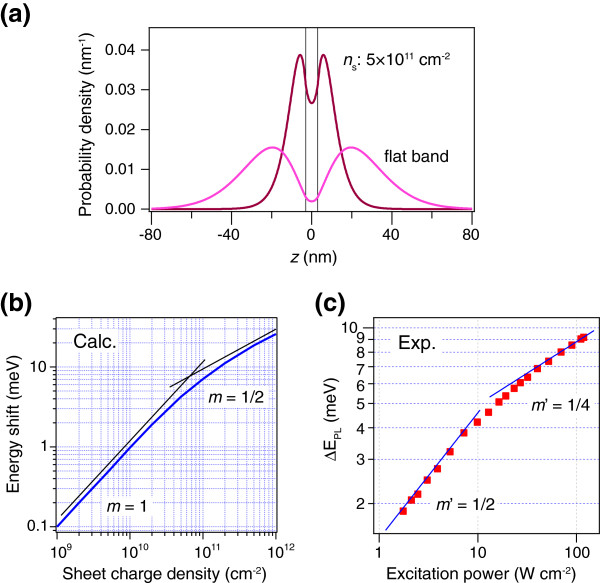
**Calculation considering excitonic effects, in comparison with the experimental results.** (**a**) Plot of the probability density for the electron under the flat band and bending band. (**b**) Double logarithmic plot of the exciton energy shift versus sheet charge density for a 6-nm GaAsSb QW. (**c**) The same data as in the inset of Figure 1, fitted with another power law.

Figure 3a plots the probability density of the electron under the flat band (*n*_s_ = 0 cm^-2^) and the bending band (*n*_s_ = 5 × 10^11^ cm^-2^). The electron probability density is calculated from the wavefunction *ψ*(*ρ*, *z*_*e*_, *z*_*h*_) by *p*_*e*_(*z*_*e*_) = ∫ _0_^*∞*^d*ρ* ∫ _− *∞*_^*∞*^d*z*_*h*_2*πρ*|*ψ*(*ρ*, *z*_*e*_, *z*_*h*_)|^2^. It is clearly seen that the electron is attracted to the well under the flat band due to the presence of Coulomb interaction. Binding energy of 3.7 meV is obtained for the exciton by comparing with the energies of the single-particle calculation Equation 1. Figure 3b shows the exciton energy shift in the GaAsSb/GaAs QW as a function of the sheet charge density. The energy shift increases linearly with the sheet charge density at the low density level, and subsequently shows sublinear dependence at the higher density regime. The power exponent at the high density regime is found to be approximately ~0.5. Although we do not have a clear explanation of the origin of the 1/2 exponent at the high power regime, the sublinear increase in the energy shift can be qualitatively understood in terms of the spatial distribution of the electron wavefunction. At a low charge density where the Hartree potential is small, most of the electrons remain in the GaAs barrier, and the electron probability density inside the GaAsSb well is negligibly small. Thus, the spatially separated charges increase in proportion to the sheet charge density, which results in a linear increase in the Hartree potential. In contrast, a significant portion of the electron wavefunction penetrates into the well at high charge density *n*_s_ = 5 × 10^11^ cm^-2^. This penetration decreases the net charge that forms the Hartree potential, leading to the sublinear increase with increasing sheet charge density.

Finally, the calculated energy shift can be connected with the experimental excitation power density, through the following rate equation

(6) $$G=B{\left(\Delta n\right)}^{2}\text{.}$$

*G* is the generation rate of the photocarrier and is proportional to the excitation power, Δ*n* is the photogenerated excess carrier density and *Β* is the bimolecular radiative recombination coefficient. Here, we ignore nonradiative recombination since the linearity of the PL intensity with the excitation power ensures the radiative dominant regime [18]. Combining Equation 6 with the numerically calculated carrier density dependence of the PL energy shift shown in Figure 3b, the following power law for the blueshift is derived:

(7) $$\Delta E_{\text{PL}}\propto \Delta n^{m}\propto G^{m'},\;m'=m/2=1/2\sim 1/4\text{,}$$

with the power factor *m’* depending on the excitation power. We show again the experimental PL peak shift in the inset of Figure 3b, along with the new power law. Transition from the low excitation regime (*m’* = 1/2) to the high excitation regime (*m’* = 1/4) is obvious. Between the two extremes, we can see the conventionally applied *m’* = 1/3 power law regime.

## Conclusions

We have analyzed the blueshift of the PL peak in a type-II QW. A one-band calculation shows that the blueshift is mainly caused by the energy shift of the confined carrier in the well. More quantitative analysis based on a self-consistent calculation including excitonic effects illustrated the transition from a linear to a sublinear increase in the blueshift with increasing sheet charge density. Combining the calculated result with the carrier rate equation, the blueshift was found to be proportional to the *m*-th root of the excitation power density, in which *m* = 1/2 ~ 1/4 and is dependent on the excitation power. The more comprehensive theory presented here predicts the 1/3-power law in the literature over a limited range of carrier density only. The above power law is consistent with the experimental results obtained from a type-II GaAsSb/GaAs QW.

## Abbreviations

MOMBE: Metal-organic molecular beam epitaxy; PL: Photoluminescence; QW: Quantum well; XRD: X-ray diffraction.

## Competing interests

The authors declare that they have no competing interests.

## Authors' contributions

MJ and MS conceived and designed the experiments. MS and SM performed the sample growth. MS conducted the optical measurements. MJ carried out the numerical calculation and drafted the manuscript. HS and HK participated in the coordination of the study. IS supervised the project. All authors discussed the results and commented on the manuscript.
